# Changes in gene expression at the early stages of barramundi (*Lates calcarifer*) muscle cell line development

**DOI:** 10.1007/s11033-026-12070-9

**Published:** 2026-06-05

**Authors:** Moran MN, Jensen SA, Jones DB, R Marcoli, Jerry DR

**Affiliations:** 1 Centre for Sustainable Tropical Fisheries and Aquaculture, College of Science and Engineering, James Cook University, QLD Townsville, Australia; 2 ARC Research Hub for Supercharging Tropical Aquaculture through Genetic Solutions, James Cook University, QLD Townsville , Australia; 3 College of Public Health, Medical and Veterinary Sciences, James Cook University, QLD Townsville, Australia; 4 Tropical Future Institute, James Cook University, Singapore, Singapore

**Keywords:** Cell culture, Gene expression, Muscle, Fish, Muscle cell differentiation

## Abstract

**Background:**

The use of cell lines in place of whole organisms can allow for the rapid and improved understanding of important cellular processes. Gaining a better understanding of muscle growth at both cellular and molecular levels allows for a better understanding of skeletal growth, gene expression, signalling pathways, and even disease mechanisms. Although there has been an increase in the number of species used to produce cell lines for research, there remains a lack of knowledge of gene expression patterns throughout early-stage cell culture starting at the muscle explant stage in a marine teleost.

**Methods:**

A muscle cell line was developed to conduct a preliminary study on the changes in gene expression throughout the early stages of muscle cell line development in barramundi (*Lates calcarifer*), an important aquaculture species. Gene expression was recorded for key muscle regulatory genes over a 14-day period in culture at the muscle explant stage, the first three passages, as well as throughout the differentiation of myoblasts.

**Results:**

The expression of major myogenic regulatory factor genes along with the ability of the cells to differentiate confirmed the cell line’s identity as a myogenic cell line. Gene expression shifted according to ongoing biological changes associated with early-stage cell culture development as well as subsequent passaging and aligned with the functional roles of the corresponding genes.

**Conclusion:**

These findings offer preliminary insight into the gene expression changes at the early stages of muscle cell line development in a marine teleost.

## Introduction

The cultivation of cell lines from piscine species has increased globally, encompassing a wide range of species and a variety of tissue types such as muscle, gill, liver, and kidney; these cell lines are documented in the online knowledge resource entitled Cellosaurus [1]. This system allows for a better understanding of cellular processes underlying growth, function, and overall health, while also offering faster results and reduced costs [2, 3]. In the past, piscine cell lines have helped facilitate the advancement of medical research by serving as a model system for various processes involved in human disease, such as gene regulation, toxicology, and carcinogenesis [3]. Recently, focus has shifted to gene editing, immunological studies, environmental stress, and disease mechanisms relevant to aquaculture species, as well as the potential of lab grown meat, with several recent examples highlighting the significance of this work and how findings from this type of research can directly inform decisions made within the seafood industry [2]. Despite advancements, there remains a lack of knowledge of gene expression patterns at the early stages of muscle cell culture starting from the muscle explant stage in a marine teleost.

When cultivating muscle cells, muscle explants are often used to provide a source of progenitor cells [4]. This method has been utilised for decades, and reduces the damage associated with primary cell isolation by maintaining the structure of the parental tissue [5]. The excising of muscle tissue into fragments mimics the effect of muscle fibre trauma, thereby triggering satellite cell migration, activation, and proliferation [6]. During muscle cell differentiation, a wide range of gene-regulatory mechanisms related to myogenesis, including gene expression changes, cell cycle exit, as well as cell fusion, all determine the fate of myoblasts [7]; therefore, differentiation is a complex and regulated process. Although some studies have looked at gene expression throughout differentiation, throughout development starting with disassociated cells, as well as throughout cell passages [7–12], there is no detailed report of the changes in gene expression throughout early-stage muscle cell culture in a marine teleost starting from primary muscle explants.

The formation of muscle cells, also known as myogenesis, is accomplished through a series of mechanisms involving the proliferation, maturation, and differentiation of myogenic precursor cells [13]. Each process can be influenced by myogenic regulatory factors, non-coding RNAs, signalling pathways, and epigenetic mechanisms, all of which have been well documented in mammalian species [14–16]. Muscle plasticity, a concept where environmental conditions can produce variable results in overall muscle structure and function, is directly influenced by myogenic regulatory factors [17]. Myogenic regulatory factors (MRF), such as myogenin (*myog*), myogenic differentiation factor 1 (*myod*), myogenic factor 5 (*myf5*), and paired box 7 (*pax7*), direct myogenic mechanisms through the regulation of proliferation, differentiation, as well as the maintenance of quiescent muscle cells [17], with each MRF being expressed in a sequential-manner [18]. For example, *pax7* is particularly known to be expressed in self-renewing cells; once the cells go through differentiation, there is usually an increase in *myod* expression and reduction in *pax7* expression [19, 20]. Another important gene influencing muscle growth, myomaker (*mymk*), is known for its control over myoblast fusion; this gene is expressed on the myoblast cell surface during fusion and becomes downregulated afterwards [21]. Alternatively, myostatin (*mstn*) negatively regulates muscle growth by inhibiting myoblast proliferation [22–25]. Since the aforementioned genes play an integral role in myogenesis and their expression throughout muscle cell differentiation has previously been described, genes related to the MRF family were included in this study to provide a preliminary description of gene expression occurring throughout the progression of cell line development as well as throughout the process of muscle cell differentiation to confirm the identity of the cell line.

Often an overlooked concept, gene expression derived from organisms or whole muscle samples does not always directly correlate to what is found within in vitro environments such as muscle cell culture. Vast differences within in vitro environments compared to standard muscle physiology found in vivo may directly influence certain pathways and/or modes of differentiation due to changes within the physical environment such as lack of structure (i.e. monolayer of attached cells) and choice of substrate [26, 27]. Another influencing factor is the absence of mechanotransduction in cell culture; detecting and responding to physical forces is fundamental to all types of cells, especially cells in dynamic tissues. Mechanical signals can directly contribute to the development and maintenance of skeletal muscle by linking mechanical forces with biochemical signals through changes in cellular and/or molecular conformations [28]. Therefore, it is essential that levels of gene expression are detailed for future studies where the expression of certain standard functional genes is otherwise assumed to be present.

Barramundi (*Lates calcarifer*), also known as Asian seabass, is the one of the largest finfish aquaculture sectors in Australia, with its popularity expanding worldwide [29]. Despite the growth and success of the barramundi industry, there remains room for improvement, and further characterisation of a model system would allow for more extensive research in the future. Although some cell lines have been established for this species [26, 30], this study focuses on reporting gene expression patterns of key genes related to the MRF family throughout the progression of cell line development as well as confirming the identity of the muscle cell line through its ability to differentiate. The aim of this study, therefore, was to describe gene expression changes occurring throughout early-stage muscle cell culture in a teleost, providing a preliminary reference for researchers to use in the future.

## Materials and methods

### Tissue isolation and myoblast cell culture

For the culture of muscle explants, a juvenile barramundi (15 g in weight) was euthanised thorough the administration of AQUI-S. Muscle tissue was immediately dissected from above the lateral line and chopped into ~ 1mm^3^ blocks. Tissue blocks were washed several times with antibiotic solution (PBS containing 500 IU/mL penicillin and 500 µg/mL streptomycin) before centrifugation at 215 x g for 3 min. Small pieces of explant tissue were inoculated into 24 well plates with Leibovitz’s L-15 growth medium (supplemented with 15% fetal bovine serum, 100 IU/mL penicillin, 100 µg/mL streptomycin, and 2.5 µg/mL amphotericin B). The muscle explants were sampled in triplicate from the 24 well plates every 2 days for a period of 14 days.

For long-term culture, muscle explants were inoculated into T25 flasks (Greiner Bio-One, Austria) to allow cells to migrate and proliferate out from the explants. After excess medium was aspirated, flasks were inverted, and 5 mL of Leibovitz’s L-15 growth medium was added to the explant-free side of the inverted flask. Flasks were transferred for incubation for 2–4 h until the tissue had adhered tightly to the substrate. The flask was then inverted once again to allow for the muscle explants to be soaked in L-15 growth medium. Immediately before the first passaging on the 14th day, a sample of muscle explant was taken from each T25 flask as a quality check in order to ensure that the conditions within the 24 well plates mirrored that of the T25 flasks; these samples were labelled as D14F.

Every 72 h, 50% of the medium was replaced until the muscle cells had reached an 80% cell confluency. Cells were passaged once they had become confluent, approximately every 2–3 days, starting on day 14. Cells were subcultured with a splitting ratio of 1:2 with 0.1% (w/v) trypsin EDTA solution (Sigma-Aldrich) to provide the best results for cell growth after passaging. Microscopy was utilised on a regular basis to observe the growth and overall status of the cells.

### PCR identification

Genomic DNA was isolated from the in vitro culture to barcode the cells to ensure they were from barramundi. For barcoding, the 18 S rRNA gene using the primers of De Santis et al. (2011) was amplified via PCR and products sent for dual direction sequencing through the Australian Genome Research Facility [23]. Sequences were then aligned to established sequences of *L. calcarifer* using Geneious Prime (Biomatters, Inc., San Diego, CA, USA) to confirm the identity of the cells. For further confirmation, sequences were inputted into BLAST and were matched with sequences that gave the best alignments within the NCBI reference database (www.ncbi.nlm.nih.gov/BLAST/).

### Differentiation of myoblasts to myotubes

Cells at passage 3 were incubated in 6 well plates with L-15 growth medium. Traditionally, muscle cell differentiation has been induced by switching from 10 to 20% fetal bovine serum to 2–10% horse serum since the medium has been shown to be more efficient for cell fusion and myotube formation [31]. Once the cells had become confluent, the growth medium was switched to differentiation medium consisting of L-15 medium supplemented with 2% horse serum, 100 IU/mL penicillin, 100 µg/mL streptomycin, and 2.5 µg/mL amphotericin B. Medium was changed every 48 h for a total of 96 h to allow for the formation of mature myotubes. Control groups were run in tandem containing the standard L-15 growth medium without horse serum. Cells were harvested for gene expression analysis and morphological observations were made every 48 h. Three replicates were carried out for each experiment.

### RNA isolation and cDNA synthesis

Muscle explants were homogenised using the Mini-BeadBeater 96 (BioSpec Products, OK USA) with 3–7 mm diameter stainless steel beads. For samples harvested from cell culture passages, cell lysates were homogenised by passing through a 20 -gauge (0.9 mm) needle attached to a syringe at least 10 times. RNeasy^®^ Plus mini kit (Qiagen) was used to extract RNA throughout different stages of primary muscle explants and cell culture development. Quality and quantity of RNA samples were measured using a NanoDrop One spectrophotometer (Thermofisher, USA) before diluting to a final concentration of 200 ng/µL. To ensure all residual traces of DNA contamination were eliminated, samples were incubated with ezDNase (Thermofisher, USA) for 2 min at 37 °C before cDNA synthesis. First strand cDNA was synthesised using approximately 2–3 µg of DNase-treated RNA with the Superscript III first-strand synthesis supermix kit (Invitrogen) with 50 ng/µL random hexamers.

### Quantification of RNA expression by quantitative real-time polymerase chain reaction (qPCR)

All qPCR reactions to report gene expression patterns of key muscle-related genes were carried out using PowerUp™ SYBR™ Green Master Mix (Thermofisher). Primers for each target can be found in Table 1. Reactions were conducted in triplicate with 2–3 ng/µL sample, each having a final volume of 10 µL. Reactions were run on QuantStudio 5 Real-Time PCR systems (Applied Biosystems, ThermoFisher Scientific). Reaction conditions were the same for all primer sets and was comprised of 2 min at 94 °C, followed by 45 cycles of denaturation at 94 °C for 30 s, annealing at 60 °C for 1 min, followed by extension at 72 °C for 45 s. A melt curve was performed at the end of the reaction to verify the specificity of the target as well as the absence of non-specific amplification. Positive controls and no-template controls were included for each assay to confirm that reactions were free of contamination. Primers for muscle-related genes were designed using Primer3 [32] and are listed below (Table 1).

**Table 1:** List of primers used in this study including α-tubulin (*α-tub*) as the reference gene, myostatin-b (*mstnb*), myogenic factor 5 (*myf5*), myomaker (*mymk*), myogenic differentiation factor 1 (*myod*), myogenin (*myog*), paired box 7 (*pax7*), and 18 S ribosomal RNA (*18s rRNA*) for species identification

Gene	F sequence	Rsequence	Product size	NCBI ref Seq
*18 S rRNA*	TGGTTAATTCCGATAACGAACGA	CGCCACTTGTCCCTCTAAGAA	94	GQ507431
*α-tub*	GGCACTACACAATCGGCAAAGAGA	TCAGCAGGGAGGTAAAGCCAGAGC	144	EU136175
*mstnb*	CACGCCATCACAGAGACAAT	GACTGGCTTGAAACTTTTGAG	115	XM_018696695.2
*myf5*	CTACGAGAGCAGGTGGAGAG	CATTCAACTGTTGCCACACC	127	XM_018661930.2
*mymk*	ACGACATTCTGGAGTACTTCAG	CCTCACAGCGATGGTTAACA	134	XM_018673725.2
*myod*	CAGGAGGACGGCTTCTACC	AGAGCTGCTGTCGTAACTTC	147	XM_018704601.2
*myog*	GGGACTGCACTACCGACC	CGTACACTGCTCTGGGGT	118	XM_018685841.1
*pax7*	ACAGTGCAAGGGTTGTCAAA	TTCTTCACTCGGAACACCTC	207	XM_018691284.2

### Statistical analysis

The transcript abundance of each target gene was calculated using the ΔΔCq method [33] with α-tubulin as the reference gene from De Santis et al. 2011 [34]. Results are shown as means ± SD. Change in expression was assessed using the non-parametric Kruskall-Wallis ANOVA. When ANOVA revealed significance, a Wilcoxon tests with Bonferroni correction were performed. Significant differences were defined by a *p* < 0.05. All statistical tests were performed in R Studio 4.2.3.

## Results

### Culture of muscle explants

**Fig. 1:**
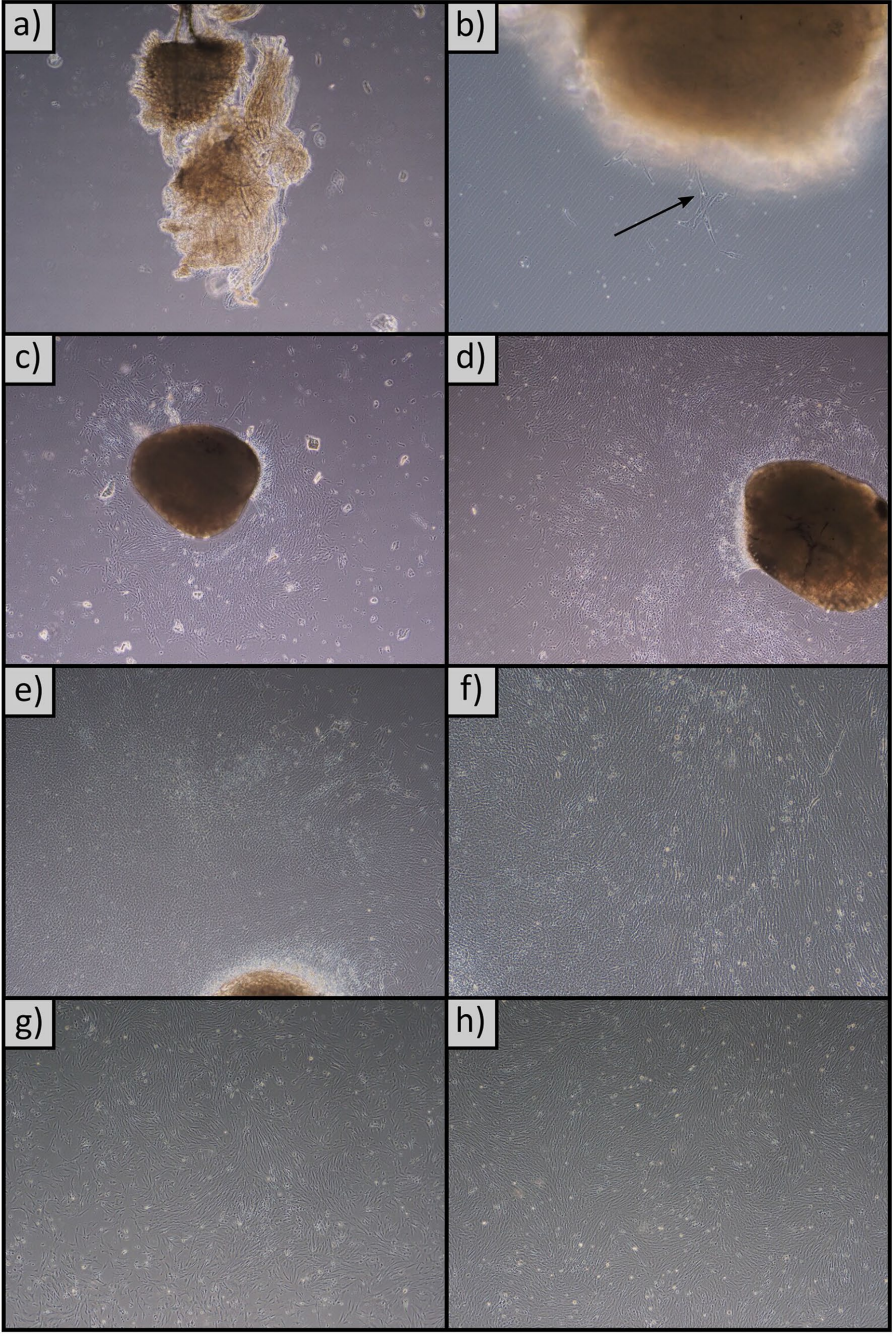
Pictures from the development of barramundi muscle cell culture: **a**) day 0, **b**) day 4, **c**) day 8, **d**) day 12, **e**) day 14, and the first three passages: **f**) passage 1, **g**) passage 2, **h**) passage 3. Arrow points to where cells were migrating out of the explant

In order to describe the changes in gene expression throughout the early stages of the barramundi cell line starting from muscle explants, small pieces of tissue were inoculated into 24 well plates with Leibovitz’s L-15 growth medium and were sampled every 2 days for a period of 14 days. The inversion of the flasks allowed for the muscle explants to become tightly adhered to the substrate on day 0 (Fig. 1a). Cells did not start to migrate out of the muscle explants until around day 4 (Fig. 1b). They then continued to proliferate and expand, and once they had become confluent at day 14 (Fig. 1e), they were passaged every 2–3 days. The passaged cells displayed the typical physical characteristics of myoblast cell culture (Fig. 1f-h).

**Fig. 2:**
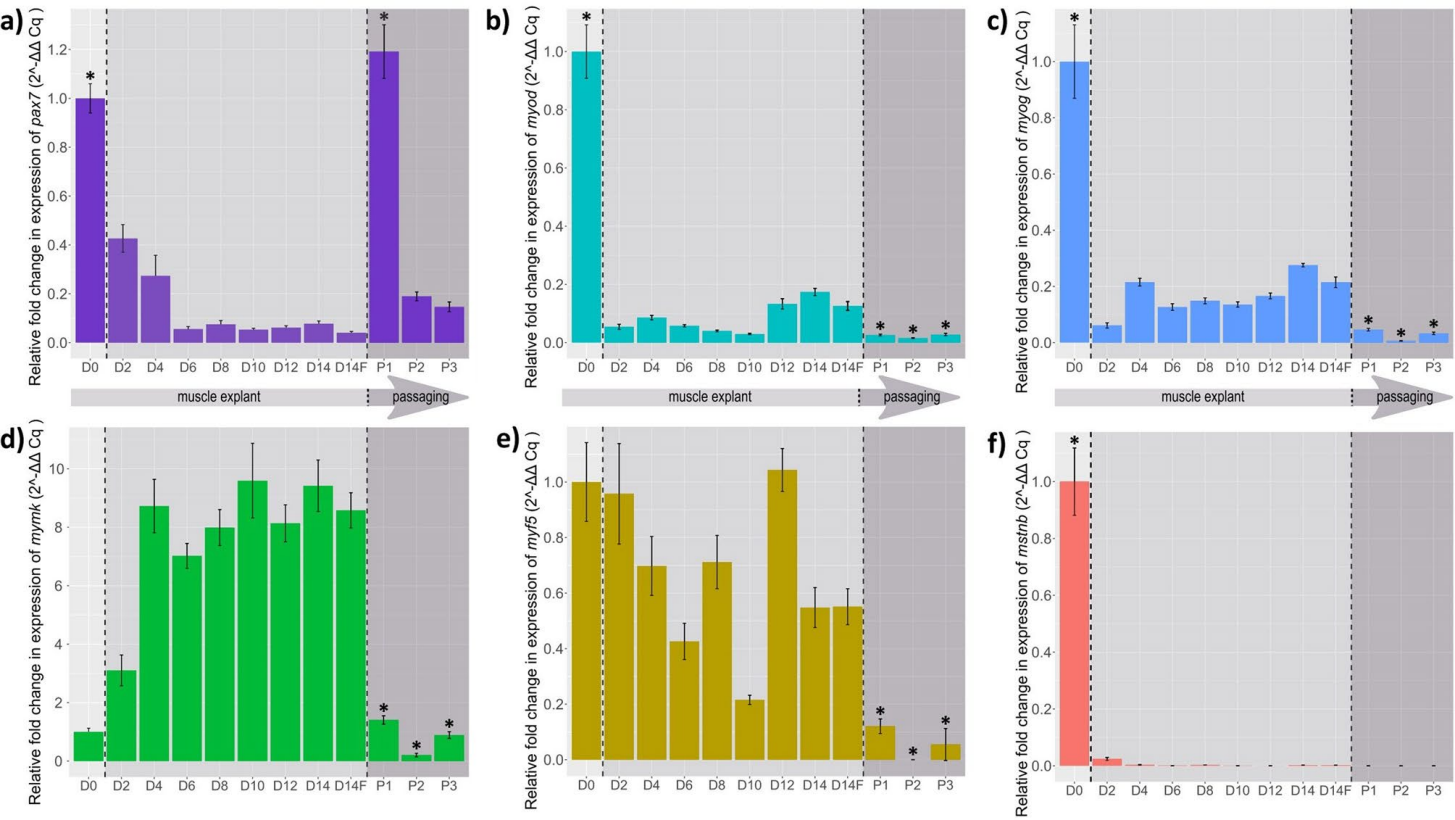
Gene expression of barramundi muscle-related genes including (**a**) *pax7*, (**b**) *myod*, (**c**) *myog*, (**d**) *mymk*, (**e**) *myf5*, and (**f**) *mstnb*, throughout the culture of muscle explants for 14 days (D representing day), and the first 3 passages (P1, P2, and P3). D14F represents muscle explants taken from the T25 flasks immediately before passaging as a direct quality control and comparison to the muscle explants cultured for the 14-day period in the 24 well plates. Wilcoxon tests were performed to compare D0 to the first 48 h of culture as a muscle explant (D2); in addition, the end of muscle explant culture (D14) was compared separately for each passage (P1, P2, P3) (* denotes p values < 0.05)

Several integral gene-regulatory mechanisms are known to drive the process of skeletal muscle growth, and their change in expression at various points in the development of the barramundi muscle cell culture is evident in these results. Throughout the period of muscle explant proliferation, targeted genes had expression upon adhesion at day 0 (Fig. 2). However, the expression of several of the myogenic-related proteins, such as paired box 7 (*pax7*), myogenic differentiation factor 1 (*myod*), and myogenin (*myog*), were observed to have significant downregulation after 48 h (*p* < 0.05). Expression then persisted at similar levels until the end of the muscle explant experiment (Fig. 2a, b, c). Out of these three genes, only *pax7* had significant upregulation of gene expression upon the first passage (*p* < 0.01), with the second and third passages having significant downregulation compared to the first passage (*p* < 0.01) (Fig. 2a). Conversely, *myod* and *myog* expression is significantly downregulated upon the start of passaging (*p* < 0.01), and each passage is significantly downregulated compared to the day before passaging (day 14) for both genes (*p* < 0.01) (Fig. 2b, c).

The expression of myogenic factor 5 (*myf5*) fluctuated throughout the progression of the muscle explant development and did not seem to present a clear pattern (Fig. 2e). In addition, expression of *myf5* appeared to be downregulated as the cells continued to be passaged and was significantly downregulated upon passaging for each passage compared to day 14 (*p* < 0.01). Interestingly, the expression of myomaker (*mymk*) was upregulated while in culture as a muscle explant, with day 4 having significantly higher expression compared to day 0 (*p* < 0.01), and then expression was significantly downregulated for each passage compared to day 14 (*p* < 0.01) (Fig. 2d). Conversely, the expression of myostatin-b (*mstnb*) was significantly downregulated (*p* < 0.01) within 48 h and expression was subsequently further reduced as time progressed (Fig. 2f). Similar levels of gene expression were observed between muscle explant samples taken at day 14 compared to muscle explants taken from each T25 flask designated for passaging at day 14 (D14F), indicating that conditions within the 24 well plates were similar to those of the T25 flasks.

### Differentiation of myoblasts

**Fig. 3:**
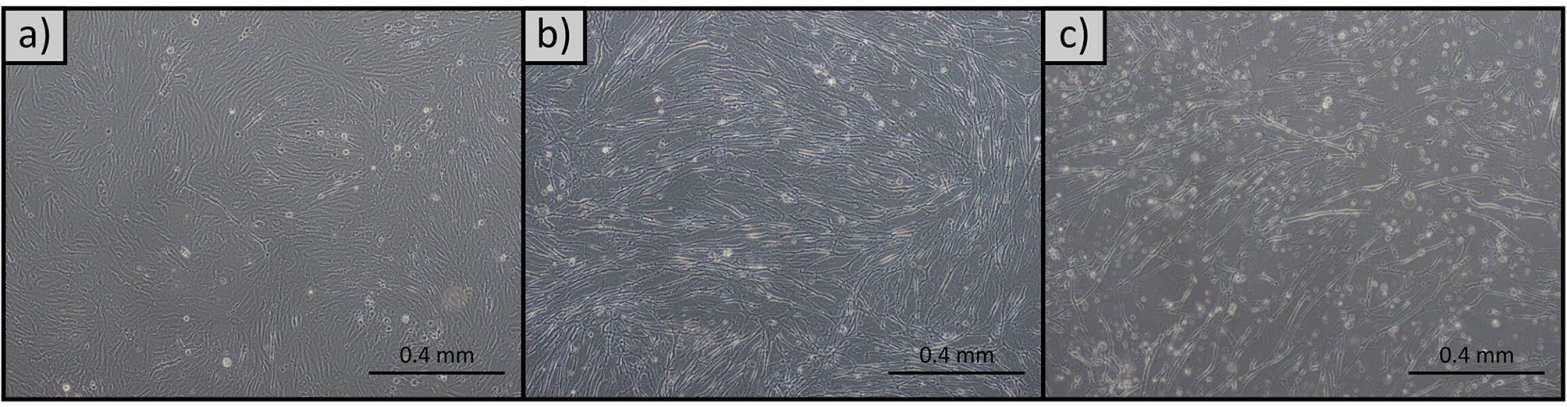
Differentiation of barramundi myoblasts into myotubes at: (**a**) 0 h, (**b**) 48 h, and (**c**) 96 h

The differentiation of myoblasts was undertaken to confirm the identity of the cell line. The myogenic differentiation of the cells was induced by incubating cells in medium containing a low concentration of horse serum. The cells displayed a high differentiation efficiency, and this was reflected in the characteristics of the cells, as well as the change of expression in differentiation-related genes. Throughout differentiation, the cells converted from their original myoblast-like appearance and formed thick myotubes (Fig. 3). This difference was evident as early as 48 h into the differentiation process.

**Fig. 4:**
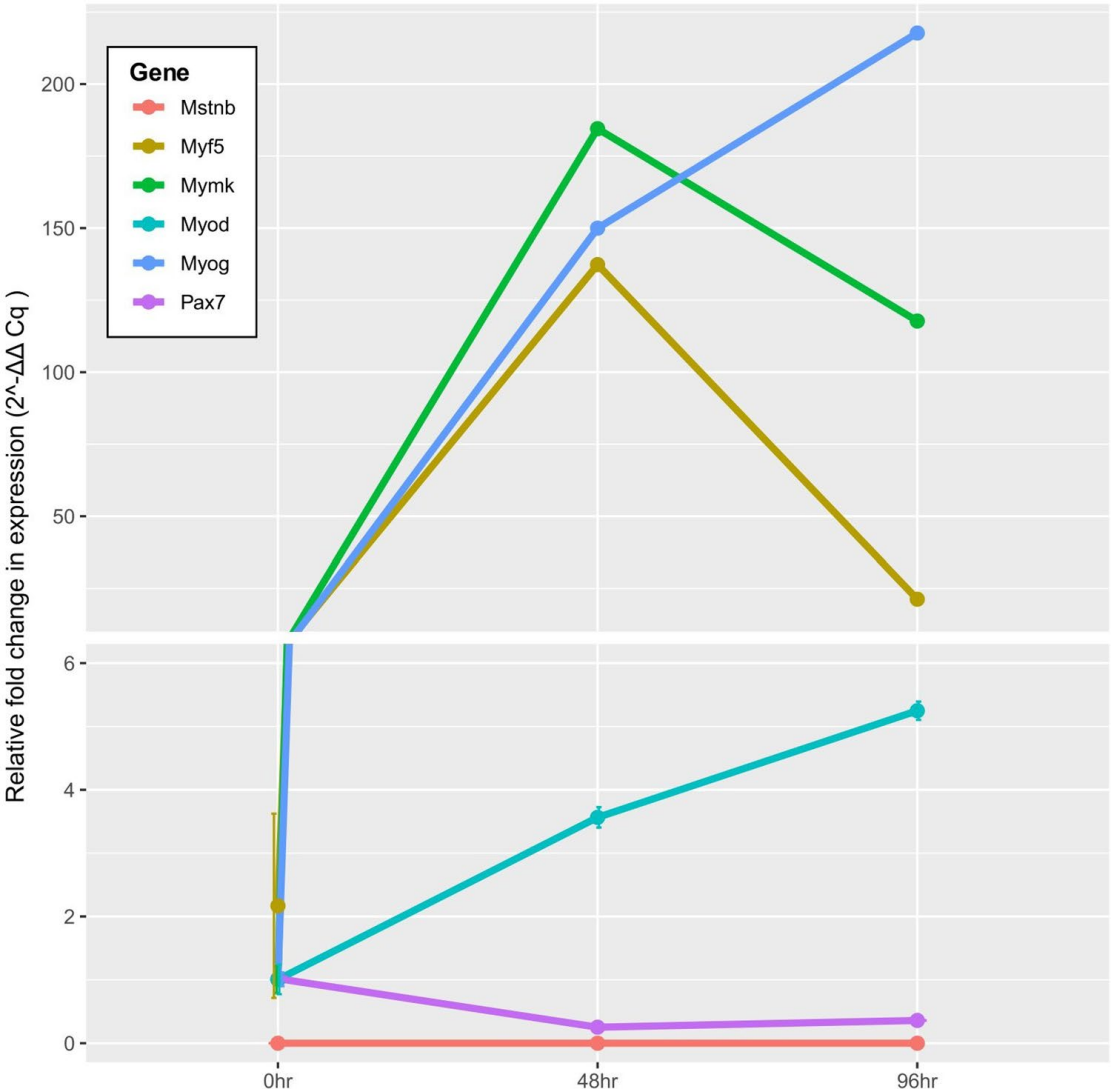
Gene expression of muscle-related genes (*mstnb*, *myf5*, *mymk*, *myod*, *myog*, and *pax7*) throughout barramundi muscle cell differentiation. Lines represent the average gene expression of each gene for every 48 h over a 96-h period with standard deviation bars included

As the cells began to differentiate, the expression of *mymk*,* myog*,* myf5*, and *myod* were significantly upregulated at the 48-h time point (*p* < 0.01) (Fig. 4). Conversely, the expression of *pax7* was observed to be significantly downregulated after 48 h (*p* < 0.01). At the end of the differentiation process, gene expression of both *mymk* and *myf5* were significantly downregulated after 96 h compared to the 48 h timepoint (*p* < 0.01). The expression of *myog* and *myod* were significantly upregulated after 96 h compared to the 48 h timepoint (*p* < 0.01), and the expression of *pax7* had similar, continued expression after the 48-h time point. Throughout the period of myoblast differentiation, no expression of *mstnb* was detected (Fig. 4).

## Discussion

Cultivating fish muscle cells can be a sustainable way to provide a reliable system for the study of skeletal growth, gene expression, signalling pathways, and even disease mechanisms [2]. However, gene expression derived from whole muscle samples does not always directly correlate to what is found within in vitro conditions. Vast differences within in vitro environments compared to standard muscle physiology found in vivo can directly influence certain pathways and/or modes of differentiation due to changes within the physical environment, such as lack of structure (i.e. monolayer of attached cells), differences in mechanical properties, choice of substrate, and choice of culture medium [26, 27]. Therefore, it is important to document changes in gene expression throughout the development of an in vitro muscle cell culture system, especially in cases where the expression of certain standard functional genes is assumed to be present.

When incorporating muscle explants for the production of a primary muscle cell culture, the expression of myogenic regulatory genes was found to change dramatically over a short period of time. The ability of skeletal muscle to acquire its regenerative capacity is derived from the presence of satellite cells [15]. These cells function as quiescent muscle stem cells. Once activated by some type of trauma, such as an injury, skeletal muscle cells start to proliferate and differentiate into myoblasts, myotubes, and are followed by multinuclear myocytes [35]. The process of self-renewal in muscle stem cells is driven by paired box 7 (*pax7*), an important transcription factor that plays a significant role in myogenesis [20]. Previously, the long-term culture of gilt-headed sea bream muscle explants resulted in the generation of new muscle fibres as original and mature muscle fibres degenerated [36], demonstrating the usefulness of incorporating muscle explants in culture to provide a source of progenitor cells [4]. In this study, *pax7* expression was found throughout the muscle explant development and steadily decreased over time, suggesting the expected degeneration of mature muscle fibres and the generation of new muscle fibres.

The myogenic regulatory factor (MRF) family is made up of genes such as myogenic differentiation factor 1 (*myod*), myogenic factor 5 (*myf5*), and myogenin (*myog*); all of these genes are able to convert a variety of cell types towards myogenesis [37]. The activation of *myod* and *myf5* are considered myogenic determination factors; they direct progenitor cells for the establishment of a skeletal muscle lineage, with their expression indicating the commitment to a myogenic lineage [15]. Expression of both *myod* and *myf5* were present throughout the explant experiment, suggesting that the cells were of myogenic lineage. The continued expression of *myf5* throughout the culture of muscle explants suggests that the satellite cells were in a proliferative state [36]. Already, *mymk* has been shown to play a role in myoblast differentiation and fusion in zebrafish [38]. As expected, myomaker (*mymk*) was highly upregulated during the muscle explant experiment and then returned to starting levels once the cells were passaged. The increased expression of *mymk* in the explant after days in culture, along with the downregulation of expression after passaging, may indicate that the presence of the explant could help aid in myoblast fusion or differentiation by providing a 3-dimensional surface or a constant source of progenitor cells, and that the cells remained as myoblasts in culture after passaging.

In this study, the barramundi cell passages displayed the typical, physical characteristics of myoblast cell culture found in previous piscine species [8, 39]. The expression of major myogenic regulatory factor genes, such as *myod*,* pax7*, and *myog*, along with the ability of the cells to differentiate into myotubes in the presence of low concentration horse serum, further confirmed the cell line’s identity as a skeletal muscle-derived myogenic cell line [11]. Myoblast cells act as a population source of self-renewing cells aiding in the homeostasis of muscle development [7]. The differentiation of myoblasts is a complex and regulated process involving the collective action of multiple genetic pathways [14, 16]. During the process of muscle regeneration, myoblasts stop their renewal process and undergo differentiation. Myoblasts differentiate into myocytes through the fusion of existing myofibers or with other myocytes to form new myofibers [19]. Through the process of differentiation, the cells transformed from their original myoblast-like appearance into thick myotubes that displayed the typical, physical characteristics of muscle cell differentiation previously found in culture from other piscine species [10, 39, 40].

Several integral gene-regulatory mechanisms have been found to drive the process of skeletal muscle differentiation. As seen in previous studies [15], *pax7* was expressed within the muscle explants as well as during the beginning of differentiation, indicating the presence of skeletal muscle satellite cells [41]. As muscle satellite cells were in the midst of their prolific phase, they generally expressed *myf5*; however, once differentiation is initiated, the expression of *myf5* falls and *myod* increases [36]. As the terminal differentiation progressed, progenitors of *pax7* began to express *myf5* and *myod*, indicating committed myoblasts and myoblast fusion. Thereafter, the increase in *myog* and *mymk* expression assisted in the formation of single-nucleated nascent myotubes [41]. In short, the barramundi muscle cells developed and differentiated similarly to previous studies [39], confirming the myogenic identity of the cell line. Therefore, this indicates that barramundi muscle cell lines may be appropriate for use in the study of skeletal growth, gene expression, signal pathways, and even disease mechanisms.

Recently, there has been great interest in cell culture research focusing on the prospects of lab-grown meat [42]. Theoretically, the interference of signalling pathways such as the downregulation of myostatin can improve proliferation and production rates, and the loss of function of myostatin has been shown to result in increased growth rate in several fish species [22]. However, unlike in mammals, the effects of myostatin in fish are not always specific to muscle but instead act as a general inhibitor of cellular proliferation and growth to control tissue mass. Most studies on the function of myostatin have been conducted in C2C12 myoblasts, with mechanisms of myostatin’s function in fish cell culture being poorly understood [43]. Similar to previous studies, the expression of myostatin-b (*mstnb*) in barramundi explants was found to dramatically drop off after being in culture for more than 48 h [36]. However, there has been documented expression of *mstnb* found in myotubes of gilthead seabream that had formed in between original fibres of the muscle explants around day 11, with the expression not increasing significantly until around 23 days, suggesting that these cells may need some type of physical scaffold in order for the cells to behave more similarly to in vivo conditions [36].

Due to the general lack of *mstnb* expression in culture, most studies investigating the role of myostatin have occurred at the explant stage and/or have induced gene expression. For example, in order to better understand the mechanisms of *mstnb* in culture, Liu et al. [44] overexpressed human recombinant MSTN (huMSTN) in Japanese flounder (*Paralichthys olivaceus*) cells and then performed gene knockdown to observe the role myostatin plays within muscle growth [44]. The need to supplement cell cultures with recombinant proteins before studying the functionality of myostatin [25, 45] suggests that expression of myostatin within culture may not be sufficient on its own for functional studies and may not impede muscle cell growth. Further research needs to be undertaken to determine whether the introduction of bio-scaffolds, mechanical stimulation, change of medium, or change in environmental conditions may affect the expression of myostatin.

Several factors must be considered before applying these findings to other species or culture conditions. Firstly, it is important to note that only a single individual was used in this study. Therefore, the gene expression documented throughout the early-stage cell culture should only be seen as a preliminary description instead of a species-wide baseline. The patterns of gene expression observed throughout muscle cell differentiation indicate that this culture is muscle-derived and exhibits functional characteristics consistent with those reported in other teleost species [39]. Since Leibovitz L-15 medium (L-15) is known to support cell growth without CO₂ and performs well at lower temperatures, many teleost cell lines established after 1994 have relied on it [3]; as a result, L-15 was selected for use in this study. The composition of cell culture medium, such as the selection of serum, buffer system, added growth factors, etc., can directly influence cellular metabolism within culture and contribute to differences between biological processes observed in vitro and those occurring in vivo [31, 46]. Accordingly, the findings of this study should not be generalised to conditions where the composition of the cell culture medium differs.

## Conclusion

To summarise, this study describes the isolation and culture of muscle cells from barramundi, allowing for future studies to have a preliminary reference of gene expression for integral genes related to the MRF family throughout early-stage cell culture. As evident in this study, genes found to have stable levels of gene expression in whole muscle samples may not show the same levels of expression in cultured cells, and their expression can also dramatically change on passaging. Therefore, this study helps fill an integral gap in the literature by providing preliminary data of gene expression within early-stage muscle cell culture where gene expression is often assumed to be similar to whole muscle samples or long-term culture conditions. Despite these differences, throughout the process of development and differentiation of the barramundi muscle cells, the overall long-term gene expression showed similarities to other muscle cell lines developed from piscine species, indicating that this system would be appropriate for use in future biological studies. In the future, a more in-depth study of gene expression under a variety of different culture and substrate scenarios may provide further knowledge towards the biological signalling pathways found within piscine cells under in vitro conditions. Collectively, this study provides a working system that allows future studies to conduct more advanced research related to integral genes within the MRF family.

## Data Availability

The raw data supporting the conclusions of this article is available on Open Science Framework (https://osf.io/v9368/overview?view_only=ee4785c09e8148459bd0d85f314d52dd).
